# Can Virtual Reality Change Minds?

**DOI:** 10.3390/bs16060865

**Published:** 2026-05-28

**Authors:** Kadir Gülcan, Ayça Demet Atay

**Affiliations:** Faculty of Communication, Near East University, 99138 Nicosia, Cyprus; ayca.atay@neu.edu.tr

**Keywords:** immersive journalism, emotion, empathy, virtual reality, journalism

## Abstract

This study investigates how immersive journalism delivered through virtual reality may shape audience responses toward refugees by activating affective and cognitive mechanisms associated with behavioral response. Drawing on four focus group sessions with a total of thirty two participants in Northern Cyprus, the research compares the empathic engagement and evaluative reflections associated with a 360 degree VR documentary with those produced through a traditional 2D viewing format. Participants who experienced the content in VR reported a heightened sense of presence, emotional proximity, and perspective taking, which corresponded with a positive change in their views toward refugees. In contrast, those who watched the same content in 2D expressed emotional discomfort yet generally did not describe a notable attitudinal shift, suggesting that non-immersive viewing maintains psychological distancing and reinforces pre-existing beliefs. The findings indicate that immersive journalism can operate as a technological catalyst for short-term attitudinal reorientation in politically sensitive contexts, particularly by eliciting embodied emotional responses that traditional formats struggle to generate. Although the study is limited by its small sample size and reliance on self-reported reflections, it contributes to the growing body of evidence that immersive media hold behavioral and perceptual relevance for journalism practice, audience engagement, and the broader public understanding of marginalized populations.

## 1. Introduction

The advent of digital technology has profoundly transformed journalism, opening new avenues for storytelling and audience engagement. Among the innovative approaches emerging in recent years, immersive journalism stands out as a powerful tool capable of promoting deeper emotional connections between audiences and news stories. Unlike traditional journalistic methods, immersive journalism places viewers within a virtual environment that recreates real-life situations, cultivating a strong sense of presence and immediacy. This enhanced sense of “being there” has been shown to elicit stronger emotional reactions, increase empathy, and potentially influence viewers’ attitudes and behavior toward complex social issues (Sánchez Laws, 2020; Shin & Biocca, 2018; Sundar et al., 2017; Rose, 2018; Hassan, 2019).

This study seeks to explore the potential of immersive journalism to enhance empathy and shape perceptions of refugees in Northern Cyprus. The research focuses on the refugee crisis in the Mediterranean and the broader Middle East. By examining how audiences respond to a VR documentary about a Syrian refugee girl living in a refugee camp in Lebanon, “My Home Shatila,” in comparison with a traditional 2D viewing, this research aims to assess whether immersive storytelling can effectively bridge emotional and perceptual gaps in a politically sensitive context. As media practices continue to evolve, understanding the capacity of immersive technology to promote social and attitudinal change remains a critical area of inquiry, holding important implications for journalism, policy development, and humanitarian communication.

## 2. Literature Review

The rapid evolution of digital media technologies in recent years has significantly transformed conventional journalism practices and reshaped the ways in which news is experienced. One of the most striking examples of this transformation is immersive journalism, which incorporates audiences into the heart of the news event by creating a strong sense of physical presence. In doing so, it shifts the audience’s role from passive viewers to active participants who experience the news. Supported by technologies such as virtual reality (VR), augmented reality (AR), and 360-degree video, this emerging form of media has gained prominence particularly in the coverage of humanitarian crises and conflict zones, where emotional engagement is high and the potential to generate empathy is critical (Grönroos & Voima, 2013; Lee & Hsiang, 2014; Lewis & Westlund, 2015).

In this context, empathy is no longer viewed merely as an emotional connection between individuals, but as a domain of political and ethical responsibility. The term “politics of empathy” refers to the ways in which audiences emotionally connect with the “other” through media representations, and how these emotional responses influence their social and political attitudes. Especially in news stories related to war, migration, and displacement, topics rich in emotional content, the capacity to enhance empathy positions the media not only as a transmitter of information, but also as a powerful agent in shaping public perception and social awareness.

Immersive journalism effectively offers a virtual world where news stories are recreated, and users are invited to an embodied experience. The main idea of immersive journalism is to allow the audience to be involved in a reconstructed scenario in the virtual world that represents the news story (De La Peña et al., 2010). Nonny De La Peña and her colleagues define immersive journalism as follows: “Producing news in a way that people can gain first-person experience of the events and situations described in the news” (De La Peña et al., 2010, p. 291). Thus, the viewers are given the feeling of “being there” and “they get the opportunity to experience the event from the perspective of a character depicted in the news story. They also have the chance to empathize with the story individually” (Çaba, 2018, p. 714).

Immersive journalism attracts the attention of the viewers because it offers them the opportunity to emotionally participate in the events depicted in the news reports (De La Peña et al., 2010, p. 298; Pantti, 2010, p. 176). Rather than treating VR solely as a state of “being there,” it should be understood as a technologically mediated simulation environment that combines immersion with interaction. It is essentially a simulation that creates virtual worlds with realistic movements or audio configurations that respond to users (Burdea & Coiffet, 2003). For example, an embodied user at the center of a VR experience becomes part of the mediated landscape and situation to such an extent that they may experience real physical reactions, such as an increased heart rate or sweaty palms, when looking over a cliff in a 3D-animated environment (Blascovich & Bailenson, 2011; Ryan Bengtsson & Van Couvering, 2022).

VR has four main elements: virtual world, immersion, emotional feedback, and interaction. The virtual world is a place where post-built content is streaming. Immersion is understood as an individual’s sense of presence, a sense of “being there” either mentally or physically. Emotional feedback and interaction are explained as the technology’s ability to respond to the actions of the users (Sherman & Craig, 2003). However, these elements are not unique to journalism; what distinguishes immersive journalism is the application of these affordances to factual storytelling and mediated representations of real-world events. Most journalism productions shown as VR today consist of 360-degree videos (Cornia et al., 2016). With 360-degree videos, audio and images can be transmitted live to the viewer, who can focus on wherever s/he wants and has the chance to observe the events from a broader angle.

Storytelling, empathy, virtual/real duality and interaction are the basic elements of immersive technologies (Soler-Adillon & Sora, 2018). In VR, the viewers are immersed in the event. By wearing VR headsets, they leave their physical environment and watch the reproduced content through this headset. A three-dimensional view and depth level is captured through 360-degree videos. By moving their head, the viewers can change the angle of view similar to the human gaze. With the use of spatial sound technology, the viewers perceive the direction of the sound as if it were real. The viewers can take a more active role in the event. They can be an observer, a character, or the main character of the story. The viewers may feel that they are in the setting of the story and react as if they were there. The sense of presence (being there) gives immersive journalism technology significant advantages over traditional narrative. It evokes a sense of more developed truth and transparency in the viewers (Benítez & Herrera, 2020; Canorea, 2022).

In a study conducted by Gallego Abellán et al. (2024), the effect of 360-degree virtual reality video on audience responses toward undocumented young migrants to Spain was examined. Of the 51 participants to the study, 23 experienced the same content in a 360-degree VR format, while 28 experienced it in a 2D format. The findings showed that the 360-degree VR experience generated stronger emotional and empathetic responses among participants. Therefore, the study demonstrates that immersive journalism may serve as an effective documentary journalism tool for communicating social issues such as migration.

As shown in Greber et al.’s (2023) study, entitled “Feeling the News? The Differential Effects of Immersive Journalism on Emotional Response,” the effects of immersive journalism on users’ emotional responses cannot be explained solely by technological inclusion; rather, they are also shaped by different conditions such as narrative form, interaction possibilities, and individual empathy tendency. The study supports the argument that VR-based journalistic experiences may generate more intense emotional and empathetic responses among audiences. Li and Lee’s (2019) examined the effects of emotional personalization strategies and presentation modality on audience experience in immersive journalism. The study included 193 participants. An experimental design was conducted based on the presence or absence of emotional personalization and whether the content was presented in VR or desktop format. The findings revealed that emotional personalization increased feelings of presence and story recall, while the VR format generated stronger feelings of empathy. In this respect, the study supports the central assumption of the present research that VR-based immersive journalism experiences may generate more intense emotional and empathetic responses among audiences.

Vázquez-Herrero (2024) also argues that immersive journalism research should move beyond technology- or device-centered approaches and adopt a story-focused perspective. Accordingly, the effects of VR-based journalistic experiences should be evaluated not only in terms of technological immersion, but also in relation to narrative structure, spatial and temporal organization, and emotional impact. The VR experience is not merely a technical tool, but also a narrative form of journalism capable of fostering presence and empathetic awareness among audiences.

### 2.1. Empathy and News Reporting

Previous research has shown that emotional participation in immersive journalism enhances empathy in the viewers; however, the extent to which this empathy translates into attitudinal change remains underexplored in the literature (Sánchez Laws, 2020).

Empathy is the ability of an individual to understand and respond to another person’s experiences (Rogers, 1983). Sinclair et al. (2017) distinguished between sympathy, empathy, and compassion, noting that empathy is characterized by features such as acting with genuine concern based on emotional understanding, assuming an altruistic role, taking action, and engaging in small acts of kindness without expecting anything in return. In essence, the emotional component of empathy involves an effective response to another person, reflecting emotional congruence.

Empathy can be described as the ability to put oneself in another person’s position and feel concern for their well-being. It involves imagining how others think and feel (Davis, 1983). Similarly, Rogers (1983) defines empathy as understanding another person’s emotions and thoughts accurately, experiencing them from their perspective, and communicating that understanding back to them. Based on the definitions provided above, this study approaches empathy as a multidimensional construct that includes both affective and cognitive components. In line with Davis’s framework, affective empathy refers to emotional resonance, compassion, and felt emotional closeness toward the suffering of others. Cognitive empathy, on the other hand, refers to perspective taking and the capacity to understand another person’s situation from her point of view. Rather than treating empathy as a single and undifferentiated response, this study uses this distinction to interpret participants’ verbal expressions of emotional reactions, empathy-based closeness, and interpretive re-evaluation during and after exposure to the news content.

The perceived distance between news audiences and news events is particularly significant, especially in the context of international news, as such stories often appear distant and irrelevant to viewers (Kwon et al., 2017). To reduce this perceived distance, many 360° journalism contents aim to emotionally and directly convey the lives of ordinary people suffering in different parts of the world. These types of content call on viewers to put themselves in the place of those who are suffering, even if they are geographically distant. The act of imagining oneself in another person’s situation through mental imagery is known as “perspective-taking” (Batson et al., 1997), and this process has been a crucial component in understanding the multidimensional nature of empathy (Davis, 1983). Immersive media extend this process beyond imagination by providing sensory cues that simulate first-person experience. When users can see, hear, and feel as another person within immersive virtual environments, experiencing the world from that person’s perspective, they tend to feel a much stronger sense of connection and “being one” with that person compared to when they are merely asked to imagine the other’s experience (Ahn et al., 2013).

In addition to the cognitive ability to process and understand the suffering of others, immersive news narratives, by presenting intense real-life situations such as the conflict in Iraq with concrete visual and auditory cues, have been found to closely simulate reality, thereby triggering emotional responses (Diemer et al., 2015; Sundar et al., 2017)

Immersive journalism technologies are thought to improve people’s empathy and are increasingly becoming a research topic (Kors et al., 2016). VR technologies have begun to be called “ultimate empathy machines”, as they strengthen the viewers’ feeling of “being there” (Constine, 2015; Milk, 2015). However, this metaphor has also been critiqued for overstating VR’s transformative capacity and underestimating contextual and individual differences. From this perspective, the effects of VR should not be interpreted as universal or deterministic. The emotional and attitudinal impact of immersive journalism may vary depending on participants’ prior attitudes, personal memories, age-related life experiences, levels of exposure to similar media content, and the sociopolitical context in which the experience is interpreted. Accordingly, the present study approaches VR not as a directly transformative medium, but as a context-sensitive experiential condition whose effects may emerge in diverse and varying ways across viewers.

Sundar et al. (2017) found that participants exposed to VR and 360° video news conditions more often articulated empathic reactions, such as sympathy and compassion toward characters in the story, than participants in text-based conditions. Studies have shown that participants in VR experiences develop empathy and compassion for the virtual characters they interact with during the experience. In addition, it has been observed that the participants may want to help these characters. Some researchers suggest using VR as a tool to reinforce pro-social behavior (Baños et al., 2004; Riva et al., 2007; Gillath et al., 2008). In a study by Sánchez Laws (2020), it was found out that news reports using immersive journalism techniques were more successful in arousing empathy than other journalism methods. With enhanced empathy, one can put oneself in someone else’s shoes, and grasp her/his feelings, and take action to mitigate her/his pain (Blank-Libra, 2016). Whether such empathic activation results in durable opinion change remains an open empirical question one that this study addresses in an exploratory manner.

#### 2.1.1. Emotions in Journalism

Visuals such as photographs, drawings, graphics, tables and moving pictures in news reports can be used to influence human attitudes, ideas and beliefs (Helmers & Hill, 2004, p. 2). The impact of visuals on people’s emotions is critical to journalism. Studies show that journalism is not only based on creating meaning, but also on sensory expectations and the experience of consuming news texts (Howes, 2006).

Along with digital journalism, when an event happens, it is often shared and discussed on social media instantly. Thus, news becomes open to interpretation on social media. In this process, emotion becomes a more important factor for both the news reporter and the consumer of news, who distributes it on social media. Through a cycle of feedback, the appeal to emotions affects the way news is produced in the future (Beckett, 2015). This feedback loop contributes to a media environment in which emotional resonance increasingly shapes news visibility and circulation.

In our age, the media have become personalized due to the increasing use of mobile devices and their constant presence in our lives. Our physical relationship with news reports has also changed, as mobile devices are always on and available to us. In addition, we integrate social networking platforms, websites and media applications into our mobile devices (Beckett & Deuze, 2016). Today, people communicate with each other through networked technologies and networked communication gives users the capacity to better understand people’s emotions and moods and is very quick at recording and evaluating human emotions. McStay (2016) calls this situation “empathetic media”. Emotions are obviously at the heart of all the foregoing developments. With emotions, people become more interactive with technology and this situation fuels people’s desire to reach news and information (Bardoel & Deuze, 2001; Heinrich, 2011; Russell, 2011; McStay, 2016). This broader shift toward affective connectivity forms an important background for understanding why immersive formats may amplify emotional processing.

Emotional journalism fits into the emotionally charged networking environment that is gaining influence today; however, it is admitted by journalists and scientists that it interferes with objectivity, which is considered a fundamental principle in journalism ethics (Peters, 2011; Beckett & Deuze, 2016). This tension between emotional engagement and journalistic objectivity constitutes a central ethical debate in contemporary media studies.

Emotions are seen by many as necessary to increase the popularity of a news story on the internet. However, the appeal to emotions in news reports is conventionally considered as mere entertainment or disturbance, reducing the value of a news report (Pantti, 2010). On the other hand, from an alternative perspective, emotions create an aesthetic atmosphere in immersive journalism and guide the viewers about how stories should be experienced (Bordwell & Thompson, 2010). These contrasting perspectives illustrate the dual role of emotion as both a narrative resource and a potential source of ethical concern.

The appeal to emotions can be an impactful tool to convey messages. Emotional messages attract the attention of viewers and viewers better understand and remember the message given in this way (Lang et al., 1995). Emotions are seen as an auxiliary narrative technique in conveying information. In addition, it is argued that emotions not only facilitate the understanding of a news report but also improve empathy in the person who reads or watches it. For example, when a person who is suffering in a difficult situation is portrayed in a news report, viewers can connect with the person who is the subject of the news report, because of which the news report can become more interesting (Grabe & Zhou, 2003, p. 316). Immersive journalism has the potential to offer viewers a deeper and richer perspective on stories, appeal to emotions, and develop empathy (Sánchez Laws, 2020). However, the intensity of emotional engagement does not automatically guarantee reflective understanding or durable attitudinal change.

Today, journalists appeal to emotions in their news coverage for several reasons. The first reason is the economic competition in the digital world. As there are many data entries in the internet environment, the information must be accurate, reliable and fast. In addition, everyone on the internet is a potential competitor. Advertising revenues of the news media constitute another economic factor. The fees that media organizations receive from advertisers vary according to the number of viewers of their news content. As a result, media organizations that present news in which emotions are used intensely receive more economic support, which may also incentivize sensationalism and emotionally charged content strategies (Bakir & McStay, 2018).

Technology comes to the forefront as the second reason for using emotions as a tool. It has been proven by researchers that appeal to emotions attracts the attention of consumers and provides more interaction (Pantti, 2010). Newspapers are primarily published on social media today rather than being sold by bookstalls. News reports appeal to emotions, because people tend to share the news contents that affect them emotionally (Jenkins et al., 2013). Digital media increases emotionality. People are less constrained in an online environment. As a result, a fertile ground is being created for the rise in media content and news contexts that aim to reveal emotional reactions (Bakir & McStay, 2018). This platform logic further embeds affective dynamics into news circulation practices.

Neuroscience comes in third place. Compared to reacting to facts, people tend to react to emotions easier. Emotions are also important for the marketing of news. It is important to know what kind of news reports people react to and how they interact with them on a personal and social level (Beckett & Deuze, 2016). These insights help explain why immersive and sensory rich formats may exert strong short-term emotional effects.

#### 2.1.2. Conflict-Sensitive Journalism, Ressentiment, and Migration Journalism in the Context of Virtual Reality

Migration journalism is produced and shaped around public spheres characterized by high levels of social tension emerging from war, political conflicts, and economic inequalities. During such critical periods, conflict-sensitive journalism positions the media not merely as a channel of information but as an actor capable of influencing the trajectory of conflict itself. The conflict-sensitive journalism approach requires a deliberate avoidance of narratives that reinforce binary oppositions, provocative language, and news discourses that reproduce exclusionary representations (Galtung, 1998; Kempf, 2007). Otherwise, decontextualized and sensationalized news content carries the potential to deepen social fears and hostilities.

As a result of such discursive processes, ressentiment may develop. Ressentiment is defined as a form of affect nourished by feelings of threat, humiliation, and injustice, constructed socially rather than individually (Nietzsche, 1997; Scheler, 2010). In media discourse, representing migrants as a “burden,” a “cultural threat,” or a “risk factor” strengthens the circulation of ressentiment in the public sphere and may pave the way for the visibility of anti-immigrant sentiment (Silverstone, 2007). Such discourses harden symbolic boundaries, in contrast to the tension-reducing and pluralistic approach advocated by conflict-sensitive journalism.

Virtual reality (VR) technology offers an alternative narrative possibility to these entrenched forms of representation. Through immersive journalism practices that incorporate users into the spatial and emotional dimensions of news events, audiences can shift from the position of an external observer to that of an experiencing subject (De La Peña et al., 2010; Sánchez Laws, 2020). In this respect, VR narratives hold the potential to develop a journalistic approach centered on contextualization and empathy rather than the surface-level imagery that fuels conflict. Thus, they may enable the temporary questioning of the abstract fears that sustain ressentiment.

Nevertheless, the impact of VR technology on public perception must be evaluated within ethical boundaries. If ethical principles and journalistic responsibility are not upheld in immersive journalism practices, risks such as the instrumentalization of suffering, the reproduction of victimhood, and the reinforcement of existing power asymmetries may emerge (Silverstone, 2007; Sánchez Laws, 2020). Therefore, VR-based journalism should be examined not solely through its emotional impact potential but within a conflict-sensitive analytical framework that interrogates the social and political conditions under which ressentiment is produced. Accordingly, this study evaluates VR’s capacity to generate short-term empathic disruptions rather than permanent attitudinal transformations within this context.

The literature outlined above provides important findings regarding the potential of immersive journalism to generate empathy. However, it offers only a limited explanation of how this empathic effect operates at the attitudinal level and to what extent it can be sustained over time. Existing studies predominantly focus on the degree to which VR intensifies emotional experience, while giving comparatively less attention to whether and how these emotional responses transform users’ established attitudes.

In areas marked by high levels of social and political tension, such as migration and refugee issues, it remains unclear whether empathy production results in genuine emotional proximity or merely leads to a cognitive and normative reassessment at a surface level. The present study emerges precisely from this gap and seeks to examine the relationship between short-term emotional intensification and the possibility of more enduring attitudinal transformation in users exposed to immersive VR experiences.

Rather than assuming VR’s capacity to generate empathy as a given, this research aims to make a critical contribution to the literature by interrogating the limits, contextual dependency, and sustainability of this empathic effect.

Previous studies have shown that immersive environments can intensify emotional engagement and enhance the perceived sense of presence. However, how this experiential intensity intersects with evaluative judgment, particularly under what conditions it can lead to a rethinking of pre-existing attitudes, has not been sufficiently clarified theoretically. The current literature largely focuses on increased levels of empathy or emotional arousal; the question of whether emotional response qualitatively transforms evaluative framing is addressed more limitedly. This distinction is particularly important in socially and politically controversial areas such as migration. Emotional impact does not imply a re-evaluation of ingrained beliefs; rather, distress, sympathy, or moral discomfort can coexist with the reinforcement of existing attitudes. Therefore, the fundamental question is not whether VR creates a stronger emotional impact, but whether the immersive sense of presence qualitatively alters the structure of evaluative engagement. This study does not proceed from the assumption that VR is a persuasive tool. Instead, the research exploratorily examines whether the sense of immersive presence functions as a mediating experiential factor that can temporarily restructure psychological distance and evaluative framing under specific contextual conditions. Therefore, the research aims to contribute to filling the theoretical gap regarding the relationship between emotional intensity and evaluative rethinking.

## 3. Methodology and Method of Analysis

This research adopts a qualitative, interpretive approach, informed by a constructionist understanding of meaning-making. Rather than seeking universalizable truths through claims of objectivity, it explores how meaning is interpreted and negotiated within specific social and cultural contexts. Interpretive methodology focuses on how individuals experience and make sense of their social world, emphasizing lived experience, language, and context. As Crotty (1998) suggests, meaning is not discovered but emerges through the interaction between subject and object. In this sense, individuals actively interpret their environments rather than passively perceive them. In contrast to positivist approaches that prioritize measurement, causality, and generalization, interpretive research seeks to understand social phenomena through the meanings people attribute to them (Denzin & Lincoln, 2005).

The study was designed as a qualitative focus group study rather than a controlled experiment. Accordingly, individual and contextual variables were not treated as factors to be isolated or statistically controlled, but as integral to the meaning-making process under investigation. Age diversity was considered during participant selection in order to capture generational differences in experience and interpretation. In addition, participants’ pre-existing attitudes toward refugees were explored prior to the screening, allowing post-viewing responses to be interpreted in relation to an attitudinal baseline. During the thematic analysis, particular attention was paid to participants’ references to war and migration experiences, everyday media exposure, and the broader sociopolitical context of Northern Cyprus. These contextual elements were analytically significant, as they shaped how participants engaged with and interpreted the immersive experience.

Focus groups, typically involving 4 to 12 participants and a moderator, are designed to facilitate interactive discussion among individuals who share certain characteristics. Rather than eliciting isolated responses, this method enables participants to reflect on and respond to each other’s perspectives, making it particularly suitable for exploring shared and contested meanings (Şahin et al., 2009; Krueger & Casey, 2000). This interactive setting can foster more open and nuanced expressions of feelings, opinions, and experiences compared to individual interviews. However, as noted in the literature, findings derived from focus groups are context-specific and not intended for statistical generalization (Hennink, 2007).

To address the research questions, the study employed thematic analysis as an interpretive analytic approach. Thematic analysis involves the systematic identification, organization, and interpretation of patterns of meaning within qualitative data (Boyatzis, 1998). Following the six-phase framework outlined by Braun and Clarke (2019), namely, familiarization, coding, theme development, review, definition, and reporting, the analysis focused on recurring expressions and interpretive patterns in participants’ accounts.

The coding process was conducted in an interpretive manner, attending to both explicit statements and underlying meaning structures. Recurring expressions related to emotional response, perceived presence, perspective-taking, and evaluative reconsideration were identified as key analytic dimensions. Expressions such as sadness, compassion, emotional closeness, fear, or feeling personally affected were interpreted as indicators of affective empathic engagement. Statements reflecting attempts to understand refugees’ experiences from their perspective, to question prior assumptions, or to revise evaluative judgments were coded as forms of cognitive empathy and attitudinal rethinking. In contrast, expressions emphasizing emotional distance, habitual exposure to similar media content, or the absence of opinion change were grouped under themes such as emotional distancing, desensitization, and attitudinal continuity. These themes were treated as interpretive analytic constructs rather than fixed or quantifiable variables.

Given the growing importance of emotional engagement in news consumption, this study explores how immersive journalism techniques shape individuals’ perceptions and attitudes toward refugees. Specifically, the study addresses two research questions:

How does immersive journalism influence empathy among viewers?

In what ways, if any, is this empathy associated with shifts in viewers’ attitudes?

In addressing the first question, empathy was examined not through standardized scales or quantitative measures, but through participants’ qualitative accounts of emotional response, perceived presence, perspective-taking, and interpretive engagement.

The study adopted a comparative qualitative design based on four focus groups. Two groups were assigned to the VR condition and two to the 2D viewing condition. Focus Groups 1 and 3 constituted the VR condition, while Focus Groups 2 and 4 served as the 2D comparison groups. Within each condition, the viewing format remained consistent: participants in the VR groups watched the same immersive video using head-mounted displays, whereas participants in the 2D groups viewed the same content in a non-immersive format. All participants were Turkish Cypriots from diverse age groups residing in Northern Cyprus.

My Home Shatila is a 4 min and 13 s 360-degree video telling the story of Fadia Alham, a 14-year-old Syrian refugee living in the Shatila refugee camp in Lebanon. The video presents Fadia’s everyday life in the camp through an emotionally driven narrative, foregrounding her fears, uncertainties, and future aspirations.

Focus group participants were recruited through snowball sampling, with particular attention paid to achieving age diversity across groups, an approach considered appropriate for exploratory qualitative research. Participants were asked semi-structured questions both before and after viewing the video, in addition to engaging in group discussions. These questions were designed to explore empathy qualitatively, drawing on the theoretical distinction between affective and cognitive empathy.

Questions such as “How did this news make you feel?”, “Did you feel as if you were there?”, and “Did the video feel real to you?” were intended to elicit verbal indicators of affective empathy, including emotional resonance, discomfort, compassion, and a sense of emotional closeness toward the subject represented in the story.

Questions such as “What drew your attention most in the video?” and “Have your opinions about refugees changed after viewing the content? If so, how?” were designed to elicit indicators of cognitive empathy. These prompts aimed to capture forms of perspective-taking, interpretive engagement, and the extent to which participants reconsidered refugees’ living conditions from the refugees’ point of view.

In this respect, the interview guide was not intended to produce a psychometric measure of empathy. Rather, it was designed to identify qualitative expressions of empathic engagement within participants’ accounts. Accordingly, the analysis focused on how participants articulated emotional identification, perceived proximity, ways of understanding others’ experiences, and potential shifts in their evaluative frameworks following exposure to either the VR or the 2D format.

### 3.1. Participants and Sampling Criteria

Data were collected using a semi-structured focus group question guide designed to elicit participants’ pre-viewing attitudes, immediate emotional responses, sense of presence, empathic engagement, and post-viewing evaluative reflections. The guide consisted of open-ended questions, allowing participants to articulate their thoughts and feelings in their own words. Rather than relying on standardized scales or fixed response categories, the study generated rich qualitative data, which were subsequently analyzed thematically.

The full question guide included the following:

Pre-viewing questions:

1. What are your general thoughts about refugees?
2. What do you think should be done regarding the refugee issue?

Post-viewing questions:

3. How did the video make you feel?
4. What drew your attention most in the video?
5. Did you feel as if you were there?
6. Did the video feel real to you?
7. How would you compare this experience with traditional news formats?
8. Did your opinions about refugees change after watching the video? If so, how?

The questions were not designed to measure empathy as a psychometric construct. Instead, they were developed to elicit qualitative expressions of affective and cognitive empathy, including perspective-taking, emotional proximity, and evaluative reconsideration following exposure to either VR or 2D news content. Accordingly, empathy is approached in this study not as a quantifiable variable, but as an experiential and interpretively articulated response.

### 3.2. Limitations

This study is based on a small, context-specific sample drawn from four focus groups conducted in Northern Cyprus. The use of snowball sampling further limits representativeness; therefore, the findings should be understood as exploratory and analytically informative rather than statistically generalizable.

The analysis focuses on immersive journalism within controlled viewing conditions (VR and 2D). It does not examine how similar content circulates across different media environments such as social media, television, or online news platforms. Given the fragmented and multi-platform nature of contemporary news consumption, audience engagement, attention, and emotional responses may vary significantly across contexts. As such, responses to VR and 2D experiences in this study should be interpreted within the specific viewing conditions in which they were produced.

Another limitation concerns temporality. The study captures participants’ immediate responses following exposure to the content but does not assess the persistence of attitudinal change over time. The findings therefore do not speak to long-term cognitive or behavioral transformation. Longitudinal and experimental designs would be necessary to explore the durability of such effects.

The study adopts a qualitative, interpretive approach to empathy. Rather than measuring empathy through standardized psychometric instruments, it examines how affective and cognitive dimensions of empathy are expressed in participants’ narratives. Accordingly, the findings do not constitute scale-based measurements but offer an interpretive account of empathic engagement in VR and 2D news experiences.

Individual and contextual differences represent an additional limitation. Although participants’ prior attitudes were explored before viewing, and relevant contextual references emerged during discussions, these were not treated as controlled variables. Instead, prior experiences, such as exposure to refugee-related media content, personal experiences of war or displacement, political orientations, and baseline empathic dispositions, are considered interpretively relevant contextual factors shaping participants’ meaning-making processes. For this reason, participants’ responses cannot be attributed solely to the immersive qualities of VR, but should be understood as emerging from the interaction between media form and participant-specific contexts.

Finally, socio-economic background was not systematically incorporated into the sampling design. Factors such as income and occupation may shape how participants interpret refugee-related content and engage with immersive media experiences. The absence of systematic consideration of these contextual dimensions limits claims to demographic representativeness and suggests that findings should be interpreted with caution.

These limitations indicate that the study does not make causal claims regarding attitude change triggered by immersive journalism. Future research could build on these findings by employing longitudinal and mixed-method designs, and by further exploring how immersive news content is experienced within the fragmented, fast-paced, and multi-platform dynamics of contemporary media environments.

## 4. Findings

The findings indicate a clear difference in levels of emotional engagement between participants exposed to the immersive VR environment and those who viewed the same content in a traditional 2D format. Participants in the VR condition more frequently articulated emotional arousal, perspective-taking, and a sense of personal identification with the person depicted in the video. These responses were not interpreted as evidence of a measurable increase in empathy, but rather as qualitative indicators of empathic engagement within an interpretive framework.

In the analysis, affective empathy was identified through participants’ expressions of sadness, fear, compassion, emotional closeness, and embodied discomfort. Cognitive empathy, on the other hand, was identified through statements reflecting perspective-taking, interpretive understanding, and the reconsideration of prior views regarding refugees’ living conditions.

This analytical distinction was used to examine how different forms of empathic engagement emerged across the VR and 2D viewing conditions, without implying a causal or quantifiable relationship between technology and empathy.

These responses suggest that the sense of immersive presence may have temporarily influenced participants’ evaluative frameworks, pointing not to a verified long-term attitudinal change but rather to a short-term emotional shift.

In contrast, participants in the non-immersive MP4 group acknowledged feelings of sadness and discomfort yet largely maintained their prior attitudes toward refugees. This pattern reflects forms of emotional habituation and desensitization that may be associated with repeated exposure to mediated suffering. This contrast suggests that traditional screen-based formats may be less likely to disrupt established interpretive frameworks within the context of a single viewing experience.

The comparative findings indicate that VR can function as a medium that intensifies emotional involvement and temporarily reduces psychological distance. The data primarily reflect immediate emotional and cognitive responses and do not provide conclusions regarding long-term cognitive restructuring.

Table 1 presents the demographic distribution of the four focus groups. Focus Groups 1 and 3 were assigned to the VR condition, while Focus Groups 2 and 4 were assigned to the 2D viewing condition. This grouping strategy was adopted not only to enable a comparison between VR and 2D viewing formats, but also to examine whether similar thematic patterns would emerge across two independent groups exposed to the same condition. Accordingly, the comparison in this study should be understood primarily as a comparison between viewing conditions rather than among four distinct group categories.

### 4.1. Participants’ Initial Attitudes Toward Refugees

Before watching the videos, participants in four focus groups were asked what they thought about refugees and what should be done regarding the refugee issue. This pre-viewing phase is important in demonstrating that participants approached the experience not from a neutral position but with pre-existing attitudinal frameworks. Prior to viewing, participants in both groups expressed similar views regarding refugees. Participants in both groups stated that the arrival of refugees would further worsen the country’s economy, reduce citizens’ welfare levels, and create difficulties in access to resources. These concerns were articulated primarily in economic and infrastructural terms rather than in humanitarian ones.

For instance, P. 5 from the first group expressed that the country was already struggling under difficult conditions and that refugees therefore constituted a threat, stating:

Our country is small; everything was built for 300,000 people. However, due to migration waves, our population has doubled, leading to high costs and difficulties in hospitals, roads, schools, and living conditions, especially rents. Northern Cyprus is only enough for us; this place is for us.

(P. 5)

For example, P. 22 from the third group stated that rents in the country have increased, the cost of living has become very high, and that the government must definitely take action on the refugee issue. P. 22 expressed the following views:

On the one hand, I feel sorry for these people; no one leaves everything behind without a reason. Life is expensive, salaries are not enough, and rents have already skyrocketed. I think there should definitely be strict limits regarding refugees. In the future, this situation could also pose the risk of taking our children’s jobs.

(P. 22)

Participants also voiced concerns that incoming refugees might lead to social conflict and an increase in crime rates. P. 16 from the second group stated that each refugee could pose a threat to the social structure:

I believe this has become a threat to the existence of society. I think it has reached a level that may cause changes, deterioration, and unrest in daily behavioral patterns, and that the seriousness of this situation is increasing.

(P. 16)

These statements indicate that many participants entered the study with pre-existing skeptical or defensive orientations toward refugees. These attitudes were framed around perceived economic burden, concerns about social stability, and competition over resources. This starting point provides a critical baseline for interpreting the emotional or evaluative shifts observed after the VR experience.

The initial comments and responses suggest that participants did not approach the viewing situation from a neutral standpoint. Rather, they brought their existing moral, socio-political, and economic interpretive frameworks into the experience. Therefore, the changes observed after viewing the video should not be understood as an isolated effect of the technology itself, but as emerging from the interaction between the immersive form and participants’ prior attitudinal orientations.

### 4.2. What Caught the Audience’s Attention in the Video

After watching the video about Syrian refugees, participants in both groups were asked the following questions: *“How did this news make you feel?”* and *“What drew your attention most in the video you watched?”* Based on participants’ narratives, it was observed that those who used the VR headset focused more closely on the environment, noticed scenes down to minor details, and described the setting in a more elaborate manner. In contrast, participants who watched the video in MP4 format stated that their opportunity to explore the spatial depth of the video was more limited and tended to focus primarily on prominent, surface-level elements. This difference is grounded in the ways participants attributed meaning to their modes of technological experience and should not be interpreted as a direct claim of causality.

*Focus Groups 1 and 3: VR Headset Viewing Condition*

In the first focus group, when participants were asked, “What drew your attention most in the video you watched?”, they often related the content to their own life experiences and discussed the scenes through references to their personal pasts. The age diversity within the group contributed to the emergence of multiple interpretive perspectives during the discussion. Participants particularly emphasized the basic and constrained living conditions depicted in the video, as well as the limited range of everyday activities shown. Visual details, such as bullet marks on the walls, were frequently highlighted as salient elements of the experience.

Notably, older participants occasionally associated the scenes with their memories of conflict periods in Cyprus, linking the viewing experience to their own biographical histories. This pattern suggests a form of empathic engagement that combines affective dimensions, reflected in emotional resonance with depicted suffering, and cognitive dimensions, reflected in the interpretation of the video through personal past experiences.

P. 5: People have no social life; they live in primitive conditions. The kitchens and homes look like ruins, and we felt that.

P. 5: We lived through similar things; the walls are full of bullet holes.

P. 8: Our homes were like this too. There are no animals on the streets—no cats, chicken, dogs, birds. There’s no greenery, no hope for the future. It’s a disgrace for our generation, having no social life and no prospects.

P. 2: People lead a simple, primitive life.

P. 6: They live under extremely primitive conditions.

P. 4: At one point, I felt as if I were in prison. (Everyone agreed here.) I wondered, “When will I be able to get out of this place?”

P. 6: It’s as though there are no humane conditions at all.

Researcher: So, did you experience the emotions directly conveyed by the video?

(Everyone responded affirmatively here.)

P. 2: It’s normal on a screen, but with VR goggles, it felt like you were actually there, as if you were living there.

P. 8: Yes, when looking through the goggles, it felt as though you were really there.

P. 19: What struck me was that I felt as if I were inside the space where that girl was. Being able to look around made the environment feel more realistic. This helped me better understand what that life might be like.

P. 21: Feeling as if I were standing in the same environment with her affected me emotionally. I noticed that there were very few things around that belonged to her. This made me think about how difficult her life must be.

The collective responses to the researcher’s question indicate that participants expressed experiencing the emotional content conveyed in the video directly. However, these statements are based on subjective narratives.

The emphasis placed by P. 2, P. 8, P. 19 and P. 21 on the sense of “being there” through the VR headset suggests that participants interpreted the experience through a perception of physical presence. Although the emphasis on intra-group agreement points to a shared perception of the experience, this should not be taken to mean that the experience occurred in the same way for all viewers.

*Focus Groups 2 and 4: 2D/MP4 Viewing Condition*

In response to the question, “What drew your attention most in the video you watched?”, participants in the second focus group highlighted several prominent elements of the video. However, unlike the first group, their responses more frequently reflected an external and observational stance rather than explicitly connecting the depicted refugee living conditions to their own personal experiences. This pattern suggests that their narratives were more often articulated within a descriptive and evaluative framework, in which the video content was discussed primarily in terms of what was seen and observed, rather than through forms of empathic identification that involve personal experiential linkage.

P. 10: The neighborhoods were slums, and it showed that the children were living in poor conditions.

P. 14: The fact that people stayed in such homes, had no electricity when it rained, and managed to survive in a damp house with little food and clothing shows how pitiful their situation is.

P. 15: What stood out to me the most was people losing their lives due to electricity.

P. 27: What drew my attention was the way the girl calmly described her daily life. She talked about what she experienced as if it were something normal. It almost seemed as though she was living an ordinary life.

P. 29: While watching, I noticed that many of the houses did not even have proper walls; most of them were unfinished, with only bare walls. That caught my attention, and there seemed to be very few signs of life around. The place where she lived looked just like the war zones we see on TV.

The prominence of the pronoun “they” in participants’ language was interpreted by the researchers as indicating the maintenance of social and experiential distance.

#### 4.2.1. Does the Sense of “Being There” in Immersive Journalism Increase Audience Empathy?

In this section, empathy is conceptualized in two related but distinct forms. Affective empathy is understood as participants’ emotional resonance with the suffering represented in the video, including feelings of fear, sadness, compassion, and emotional closeness. Cognitive empathy, in turn, is understood as processes of perspective-taking and attempts to understand the lived reality of the refugee subject beyond surface-level observation.

Participants who used VR reported a strong sense of “being there” while watching the video. In particular, participants who had experienced the period of intense intercommunal violence in Cyprus between 1963 and 1974 appeared to draw on their own memories of war and displacement when engaging with the video. This suggests that the immersive experience may have interacted with autobiographical memory, potentially activating forms of emotional recall.

One 70-year-old female participant (P. 8) stated that the video frightened her and reminded her of the war in Cyprus:

I felt there, as if I was in the video. I had war like this in the past. I was a little scared when I watched the video. For a moment, I felt as if I was going to experience the same things again. So frankly, I felt sorry for them.

(P. 8)

Watching the video with a VR headset, participant 4 also said that the video reminded him of the migration period and the war in Cyprus in the past:

We actually experienced migration somewhere. I was born in the south. We have experienced similar things as well. There was a war. We went to the refugee camps in 1974. We stayed in the camps, and then they took us to Türkiye. We were accepted as refugees there, that is, we were refugees, and then we moved from south to north [of Cyprus]. I can feel very well what these people are going through and, of course, I don’t wish anyone to experience such days.

(P. 4)

P. 4, who experienced the video through a VR headset, stated that it made him feel as though he were truly present in that environment. He also emphasized that VR technology should be utilized in schools, suggesting that it might dissuade those inclined toward war from pursuing it:

After the video, of course, I felt like I was there. I felt like I lived there, and I think this technology, this video should be used in education as well. I wish schools would show videos with this technology and people would see what war is and sense it. I don’t think anyone watching a video with this technology would desire war any more.

(P. 4)

P. 24, the 73-year-old female participant who experienced the video through a VR headset, stated that the video she watched using VR reminded her of the war on the island.

When I looked around with the headset, I felt how empty and cold the place where that child lived was. Then, when I turned my head, I noticed bullet marks in some corners, and this disturbed me greatly and took me back to my youth. Since we witnessed the war in Cyprus at that time, there were bullet marks in some places, and it reminded me of those memories.

P. 23, the 53-year-old female participant who experienced the video through a VR headset, female participant stated that after watching the video in VR, she associated the character in the video with her own childhood and empathized with the character.

What affected me the most was feeling as if I were sharing the same space with her. It was as if I had lived through the same period with her in my childhood. The war there reminded me of my own childhood. Normally, the things I watch remain in my mind, but here it felt as if my body also reacted.

These accounts suggest that the VR experience was not only a visual mode of viewing, but was often described by participants as an emotionally intense experience connected to the reactivation of past memories. Analytically, such responses indicate affective empathy, expressed through feelings of fear, sadness, and emotional closeness, as well as cognitive empathy, as participants interpreted the refugee experience through their remembered histories of war, displacement, and vulnerability.

Participants who watched the video in MP4 format emphasized empathy less than VR viewers. For example, P. 10 who watched the video in MP4 format, stated that he had watched many videos in this format and on the subject before. He stated that these videos are a “fact of the life”:

I’m upset, but I’ve watched a lot of videos like this. I felt sorry for all of them before, but I also feel sorry for myself and my own people, I feel sorry for us, even more so. Therefore, the priority should be our own people. First, those who live in this country… Like I said, I’ve watched a lot of videos like this before. Of course, this video is not ordinary, these are the reality of the world, the life of refugees, they are real, but, ultimately, we must think about ourselves. So, in the end we must think about ourselves. Frankly, this video did not affect me much.

(P. 10)

Participants who watched the video in MP4 format expressed less empathy compared to those who viewed it in VR. For example, P. 29, who watched the video in MP4 format, stated the following:

Yes, the video we watched was emotional, but to be honest, it did not affect my feelings very much. We already see this kind of footage every day in the news.

This account was interpreted as an example suggesting that repeated exposure to media representations may lead to emotional desensitization, which in turn can limit the intensity of empathic responses. Therefore, the difference observed here appears to be related not only to the technology itself but also to viewers’ prior media exposure and personal histories.

The data suggest that empathic engagement in the VR condition did not emerge uniformly across all participants. Rather, it appeared to be more pronounced among participants whose memories of war, displacement, or insecurity resonated with the scenes depicted in the video. In this sense, the immersive experience was not shaped solely by technological affordances, but also by participants’ personal histories and lived experiences.

*Focus Groups 1 and 3: VR Headset Viewing Condition*

Regarding the feeling of “being there,” participants in the first group generally responded positively to the question, “Did you feel as if you were there?” During the discussion, they shared emotional expressions such as “I wanted to reach out and hold her hand” and “I wanted to hug her.” It was evident that participants responded to this question in a personal tone, expressing strong emotional reactions. The frequent presence of the girl in the video had a significant emotional impact on them.

P. 8: Yes, it felt as if she was standing right in front of you, looking at you.

P. 6: I felt as if I was having a conversation with her.

P. 8: Yes, the girl had such a warm look.

P. 3: This made the video feel more emotional, as if you were inside it—she appeared everywhere, almost as if she was really there.

P. 5: At one point, I even felt uneasy, as if she might be watching me while sitting on a rock.

P. 2: Good thing I didn’t actually reach out and hold her hand.

P. 6: I was close to reaching out and hugging her.

P. 17: Yes, honestly, I really felt as if I was there. Especially because I could look around, it did not feel like just watching; it felt like being inside that environment. The girl was right in front of me and it felt as if she was talking to me.

P. 20: I felt something similar. It was not like a normal video because I was not just looking from the outside. It felt as if I was inside the same environment where the girl was. This made the experience feel more real and more intense.

P. 21: Especially the moment when I felt that I was standing inside that room, I felt very strange. For a moment, I actually did not want to be there. It was as if I could feel the weight of that environment on me. It was not just seeing a video; it felt like living there at that moment.

These accounts suggest that the VR experience generated not only a spatial sense of “being there” but also a perception of interpersonal proximity. Participants’ expressions of a desire to establish physical contact (such as reaching out a hand or embracing) indicate that the viewing experience shifted from passive observation toward a form of encounter that was felt in an embodied manner. However, this should be interpreted not as a genuine interaction but as an intensified mode of perceptual and emotional engagement.

Furthermore, the young girl’s direct gaze toward the camera may have strengthened the viewer’s sense of social presence. This element is significant in demonstrating that empathic responses are shaped not only by the content itself but also by the mode of representation.

*Focus Groups 2 and 4: 2D/MP4 Viewing Condition*

Participants who did not use VR headsets generally responded to the focus group question, “Did you feel as if you were there?” by stating that the video did not create a true sense of immersion for them. While they acknowledged the emotional and depressing nature of the content, they described the experience as similar to watching ordinary online videos, lacking the powerful feeling of being physically present in the scene.

P. 9: I’ve watched a lot of videos like this, and these types of videos are very popular right now, so for me, it wasn’t very different from the other videos I’ve watched.

P. 15: It didn’t feel real, but I did get a bit of a sense of helplessness from the video. Other than that, it didn’t give me much of a sense of reality.

P. 16: I didn’t get a sense of reality either, but I still wouldn’t want anyone to live in such an environment.

P. 13: I didn’t exactly feel like I was there. There were things in the video that caught my attention, and I think it was a sad video, but I didn’t fully get the feeling of being there.

P. 14: I didn’t feel like I was there either. The video was a bit emotional and upsetting for me, but I didn’t feel as if I was actually there.

P. 12: I didn’t get that feeling either, to be honest. It felt like the regular videos I watch on YouTube.

P. 30: It felt like the other videos I normally watch. Yes, it was a sad video, but there are many videos like this. So, for me, it was not really different from the others.

These statements make visible the distinction between emotional response and the sense of presence. Although participants were affected by the content, this impact largely remained at the level of cognitive awareness and sadness; an experience in which they positioned themselves within the scene did not emerge. This suggests that empathic response deepens when accompanied by a sense of embodied and spatial involvement, whereas in the absence of this dimension, the emotional effect remains more limited and surface-level.

Moreover, participants’ comparison of the experience to “videos on YouTube” indicates that the mode of viewing was perceived within the same category as everyday media consumption practices. This suggests that, despite the dramatic nature of the content, the mode of representation was able to situate the viewer within the framework of an ordinary media experience. From this perspective, the findings suggest that prior exposure to similar media content may have influenced participants’ interpretive distance and emotional engagement. The differences observed between the VR and 2D conditions should therefore be understood not only in relation to format, but also within the broader context of media exposure patterns and experiential familiarity.

#### 4.2.2. How Did Empathic Engagement Relate to Attitudinal Reconsideration?

After watching the video, participants in four focus groups were asked, “Have your opinions about refugees changed after viewing the news content? If so, how?” Prior to the study, it was anticipated that the immersive qualities of VR might encourage heightened empathic engagement and, in some cases, support evaluative reconsideration. The findings partially align with this expectation.

Participants in the VR condition more frequently articulated stronger forms of empathic engagement and, in several cases, described a shift toward more positive reconsideration of their attitudes toward refugees. In contrast, participants in the MP4 condition did not report comparable shifts in their evaluative positions. As noted earlier, participants in the MP4 group also reported prior exposure to similar media content. While they expressed emotional discomfort in response to the video, their evaluative orientations toward refugees appeared to remain largely stable.

This difference indicates that emotional arousal alone is not sufficient to produce attitudinal transformation. Such transformation appears to emerge when emotional engagement is combined with a strong sense of “being there” (presence). In this context, VR functions not merely as a tool that intensifies emotion but as a medium that relocates the viewer from an external observer position into the experiential field itself, thereby reconfiguring the experiential conditions under which moral and evaluative judgments are formed.

Among the eight participants who watched the video using VR headsets, six had negative opinions before watching the video, and their views on refugees changed after viewing, as shown in Table 2. Two participants, on the other hand, held positive opinions about refugees from the beginning and did not experience any change in their views after watching the video. In the case of participants who watched the video without the headset, none of the eight participants showed any change in their initial opinions, indicating that they maintained the same views even after watching the video.

These findings indicate that immersive experience can trigger a short-term process of evaluative reconsideration, particularly among individuals who initially held negative attitudes. However, the absence of change among participants who already held positive attitudes from the outset suggests that VR does not function as a universal persuasive tool. Rather, it operates as a context-dependent catalyst whose effects are shaped by the viewer’s existing emotional and moral positioning.

In Table 2, the categories “positive” and “negative” do not represent measurements derived from a Likert-type scale or standardized attitudinal instruments. Instead, they function as interpretive summaries of participants’ evaluative orientations as expressed in their open-ended responses during pre- and post-viewing discussions. These categories reflect analytical constructions rather than fixed psychological states. Ambivalent or mixed expressions were interpreted within the broader narrative context of each participant’s account, with attention to tone, framing, and evaluative direction.

P. 2, who watched the video with a VR headset, stated that his views on refugees were negative before watching the video, but after watching it, he empathized and took a positive perspective.

In fact, our problem is not with refugees, the reason for our concerns is actually political. Our views are a bit antipathetic about refugees, but after the video, I feel like we should view the situation a little more emotionally. I think a little more positively about refugees now after watching the video.

(P. 2)

P. 18, who watched the video with a VR headset After watching the video with the VR headset, the participant stated that their views about refugees changed in a positive direction and that the experience left a lasting impression on them.

A much heavier feeling emerged inside me. After this experience, the issue of refugees became more real for me. It no longer feels like just a piece of news, but like a life that is actually being lived. This video left a lasting impression on me.

P. 6, who watched the video with a VR headset, stated that her feelings were already positive before watching the video, but after watching the video she thought that the refugees needed help.

So, I never had negative thoughts about refugees. People leave their country, for all kinds of reasons, political reasons, all kinds of wars, racial…I have never found them strange. Nobody wants to leave their country. After the video, I think they need help. They need to go to another country as soon as possible. No person should live under these conditions, this feeling was also conveyed in the video.

(P. 6)

P. 3, who watched the video with a VR headset, stated that the video changed his perspective on refugees, “although not drastically.”

When something visual is involved, people feel more emotional, especially when you see it in front of you, when you are directly in it. For this reason, I believe that the video made a change in my thoughts, although not too extreme. We feel pain when we see it like this, it affects us more when it is a visual source. No one wants to be in the place of these people. Nobody wants to be in this situation, especially after the video.

(P. 3)

P. 5, who watched the video with a VR headset, stated that “his emotions have changed.”:

My emotions have, of course, changed. As someone who has lived through these in his own time, as someone who has seen war, I suddenly put myself in their shoes, and what I went through in those days is what I see now. It is difficult to live in these conditions. I think this is the shame of the age. At the moment, I think more positively than before.

(P. 5)

Watching the video with a VR headset, P. 7 also stated that the video she watched changed her thoughts and emphasized that she felt people’s pain:

Of course, it did [change my feelings]. I was more upset. When I saw it, my thoughts changed, it made me emotional.

(P. 7)

Contrary to the participants in the first focus group, the second focus participants expressed no opinion change after watching the video. P. 11 stated that his views have not changed:

Of course, after watching the video, my thoughts did not change.

(P. 11)

P. 19, who watched the video with the VR headset, stated that his previously negative views about refugees changed in a positive direction. He also expressed that this experience helped him realize how difficult the lives of refugees are.

If I had watched it on a normal screen, I would not have felt this much emotion. This experience made me feel more realistically how difficult their lives are. It was not just watching; it felt as if I had actually lived that moment.

P. 29, who watched the video in MP4 format, noted that her previously negative views remained negative. Although she acknowledged that the video was sad, she emphasized that its emotional nature did not lead to any change in her views.

Another participant who watched the video in MP4 format, P. 10, also indicated that he was upset about the video content he watched, but that the video was not effective enough to change his mind.

I’m still thinking the same way. There has been a change in my feelings, but my opinion has not changed, even though I am sad, I think the same. Frankly, it wasn’t an effective video to change my mind.

(P. 10)

Along similar lines, P. 12 who watched the video in MP4 format remarked that the video made him sad; however, his opinions did not change:

Yes, the video made me sad. I can’t say it didn’t hurt, but there are a lot of videos like this around, so we feel sorry for all of them, but if I am to talk specifically about this video, it didn’t change my mind. Because as I said, this situation is starting to hurt us now.

(P. 12)

## 5. Conclusions

Although limited in scope, the findings of this study are broadly consistent with previous research suggesting that VR may, under certain conditions, foster empathic engagement. Participants who watched the video through VR headsets generally reported higher levels of emotional engagement and a stronger sense of presence compared to those who viewed the same content in 2D format. This aligns with prior studies (Baños et al., 2004; Riva et al., 2007; Gillath et al., 2008; Sánchez Laws, 2020), which suggest that immersive journalism can facilitate more affectively engaged forms of audience reception. However, the present study does not allow for conclusions regarding the durability or generalizability of these patterns.

Across the focus groups, VR participants more frequently described a sense of “being there,” accompanied by references to environmental details such as bullet marks on walls and power outages. The 360-degree format also encouraged exploratory visual attention across different spatial directions. In some cases, participants reported strong emotional reactions, including crying.

Importantly, the findings suggest that empathic engagement was not solely a function of technological immersion, but was also shaped by participants’ personal histories. In particular, individuals who had lived through the Cyprus conflict (1963–1974) appeared to relate the immersive experience to their own memories of fear, displacement, and insecurity. This indicates that immersive media may interact with autobiographical memory in shaping emotional and interpretive responses.

In the second and fourth focus groups (MP4 condition), participants who reported positive attitudes toward refugees prior to viewing generally maintained similar evaluative orientations after watching the video. Many participants described the proliferation of similar media content as contributing to a sense of normalization, in which representations of human suffering were perceived as “part of everyday news.” Compared to VR participants, MP4 viewers tended to focus less on specific visual details within the video and more on its general informational content.

Overall, the findings should not be interpreted as evidence of lasting or generalizable attitudinal change. Rather, they point to short-term emotional responses and momentary forms of evaluative reflection. Empathy and attitudinal orientations were not measured through standardized instruments or closed-ended scales, but were instead interpreted through participants’ narrative accounts. Accordingly, the findings reflect qualitative patterns of meaning-making rather than quantifiable changes in empathy or attitude.

The results suggest that VR may, under certain contextual and experiential conditions, facilitate heightened empathic engagement by strengthening participants’ sense of presence. However, this effect is neither uniform nor independent of prior media exposure and personal histories. In particular, the sociocultural context of Northern Cyprus, where memories of displacement and conflict remain salient, may have shaped participants’ interpretive and emotional responses.

These findings contribute to ongoing discussions in immersive journalism by highlighting that emotional and evaluative responses are co-constructed through the interaction between media format, prior experience, and autobiographical memory. Rather than producing direct or uniform effects, immersive technologies appear to open a space for differentiated forms of engagement.

From a broader perspective, the study suggests that immersive journalism should not be understood as a deterministic tool for empathy production, but rather as a contingent communicative environment that interacts with users’ lived histories and interpretive frameworks. This has important implications for journalistic practice and media ethics, particularly in relation to the representation of human suffering in immersive formats, and the potential risks of emotional standardization or desensitization through repetitive exposure. Overall, the findings indicate that immersive journalism does not produce uniform empathic or attitudinal outcomes, but instead facilitates differentiated forms of emotional engagement shaped by the interaction between media format, autobiographical memory, and prior media exposure.

Future studies should prioritize longitudinal, and where possible mixed-method or experimental designs, to examine the long-term sustainability of immersive journalism’s effects and to explore how these vary across individuals with different prior experiences, media exposure patterns, and biographical histories of conflict or migration.

## Figures and Tables

**Table 1:** Age and Gender Distribution of Focus Group Participants.

Focus Group 1 Participants Using the Headset (Participant No.)	Gender	Age	Focus Group 2 Participants Not Using the Headset (Participant No.)	Gender	Age
P. 1	M	27	P. 9	M	29
P. 2	M	31	P. 10	M	33
P. 3	M	35	P. 11	M	38
P. 4	M	45	P. 12	M	45
P. 5	M	57	P. 13	F	33
P. 6	F	42	P. 14	F	38
P. 7	F	54	P. 15	F	41
P. 8	F	70	P. 16	F	72
**Focus Group 3** **Participants Using the Headset (Participant No.)**	**Gender**	**Age**	**Focus Group 4** **Participants Not Using the Headset (Participant No.)**	**Gender**	**Age**
P. 17	M	29	P. 25	M	27
P. 18	M	31	P. 26	M	34
P. 19	M	36	P. 27	M	36
P. 20	M	48	P. 28	M	48
P. 21	F	32	P. 29	F	30
P. 22	F	42	P. 30	F	47
P. 23	F	53	P. 31	F	57
P. 24	F	73	P. 32	F	75

**Table 2:** Interpretive Coding of Participants’ Pre- and Post-Viewing Attitudes Based on Open-Ended Responses.

Focus Group 1 Participants Using the Headset (Participant No. & Gender)	Age	Before	After	Focus Group 2 Participants Not Using the Headset (Participant No. & Gender)	Age	Before	After
P. 1 M	27	Negative	Positive	P. 9 M	29	Negative	Negative
P. 2 M	31	Negative	Positive	P. 10 M	33	Negative	Negative
P. 3 M	35	Negative	Positive	P. 11 M	38	Negative	Negative
P. 4 M	45	Positive	Positive	P. 12 M	45	Negative	Negative
P. 5 M	57	Negative	Positive	P. 13 F	33	Negative	Negative
P. 6 F	42	Positive	Positive	P. 14 F	38	Positive	Positive
P. 7 F	54	Negative	Positive	P. 15 F	41	Positive	Positive
P. 8 F	70	Negative	Positive	P. 16 F	72	Positive	Positive
**Focus Group 3** **Participants Using the Headset (Participant No. & Gender)**	**Age**	**Before**	**After**	**Focus Group 4** **Participants Not Using the Headset (Participant No. & Gender)**	**Age**	**Before**	**After**
P. 17 M	29	Negative	Positive	P. 25 M	27	Negative	Negative
P. 18 M	31	Negative	Positive	P. 26 M	34	Positive	Positive
P. 19 M	36	Negative	Positive	P. 27 M	36	Negative	Negative
P. 20 M	48	Negative	Positive	P. 28 M	48	Positive	Positive
P. 21 F	32	Positive	Positive	P. 29 F	30	Negative	Negative
P. 22 F	42	Negative	Positive	P. 30 F	47	Negative	Positive
P. 23 F	53	Positive	Positive	P. 31 F	57	Positive	Positive
P. 24 F	73	Negative	Positive	P. 32 F	75	Negative	Negative

## Data Availability

The data used to support the findings of this study are available from the corresponding author or first author upon request.
